# Aging-dependent DNA hypermethylation and gene expression of GSTM1 involved in T cell differentiation

**DOI:** 10.18632/oncotarget.18109

**Published:** 2017-05-23

**Authors:** Shu-Hui Yeh, Cheng-Ling Liu, Ren-Chieh Chang, Chih-Chiang Wu, Chia-Hsueh Lin, Kuender D. Yang

**Affiliations:** ^1^ Graduate Institute of Long-Term Care, MacKay Medical College, New Taipei City, Taiwan; ^2^ Department of Medical Research and Development, Show Chwan Memorial Hospital at Chang Bing, Changhua, Taiwan; ^3^ Department of Medical Research, Kaohsiung Chang Gung Memorial Hospital, Kaohsiung, Taiwan; ^4^ Institute of Clinical Medicine, National Yang Ming University, Taipei, Taiwan; ^5^ Department of Medical Research, Mackay Memorial Hospital, Taipei, Taiwan

**Keywords:** aging, epigenetic, DNA hypermethylation, T cell differentiation, GSTM1, Gerotarget

## Abstract

This study investigated whether aging was associated with epigenetic changes of DNA hypermethylation on immune gene expression and lymphocyte differentiation. We screened CG sites of methylation in blood leukocytes from different age populations, picked up genes with age-related increase of CG methylation content more than 15%, and validated immune related genes with CG hypermethylation involved in lymphocyte differentiation in the aged population. We found that 12 genes (EXHX1、 IL-10、 TSP50、 GSTM1、SLC5A5、SPI1、F2R、LMO2、PTPN6、FGFR2、MMP9、MET) were associated with promoter or exon one DNA hypermethylation in the aged group. Two immune related genes, GSTM1 and LMO2, were chosen to validate its aging-related CG hypermethylation in different leukocytes. We are the first to validate that GSTM1_P266 and LMO2_E128 CG methylation contents in T lymphocytes but not polymorphonuclear cells (PMNs) or mononuclear cells (MNCs) were significantly increased in the aged population. The GSTM1 mRNA expression in T lymphocytes but not PMNs or MNCs was inversely associated with the GSTM1 CG hypermethylation levels in the aged population studied. Further studies showed that lower GSTM1 CG methylation content led to the higher GSTM1 mRNA expression in T cells and knockdown of GSTM1 mRNA expression decreased type 1 T helper cell (Th1) differentiation in Jurkat T cells and normal adult CD4 T cells. The GSTM1_P266 hypermethylation in the aged population associated with lower GSTM1 mRNA expression was involved in Th1 differentiation, highlighting that modulation of aging-associated GSTM1 methylation may be able to enhance T helper cell immunity in the elders.

## INTRODUCTION

Aging is a multifactorial process that results in a progressive loss of regenerative capacity and tissue functionality. Public interest in aging has increased not only in longevity but also prevention of age-related physical disability or immunodepression [1]. There is accumulating evidence that aging is developmentally regulated by gene-environment interaction. For instance, smoking exposure is associated with epigenetic modifications for aging [2], and putatively related to the oxidative stress [3].

Epigenetics refers to chromosome changes that do not modify the genetic code, but influence its expression and function in fetal stage and afterbirth. Among epigenetic modifications, DNA methylation is best characterized, which in mammals occurs primarily at 5′-cytosine of CpG islands, and are often located in the promoter or exon 1 regions of many genes and involved in transcriptional regulation [4]. Previous studies have revealed that total number of altered methylation sites increases with increasing age, and that they could serve as markers for aging [5].

DNA hypermethylation is involved in various developmental pathological phenomena and even diseases in adult life [6]. The DNA hypermethylation in the promoter region of GSTM1 accompanied by a consequent decrease of correspondent mRNA level, has been implicated in oxidative stress and macular degeneration [7]. Oxidative stress has been shown to influence metabolism of experimental eye lenses [8], and GSTM1 genotypes have also been implicated in the pathogenesis of age-related cataracts [9].

In addition to macular degeneration and cataracts, aging-related oxidative stress and inflammatory reaction have been implicated in the pathogenesis of experimental intervertebral discs [10], outcome of traumatic hip injury repairs [11] and degenerative diseases [12, 13] in the elderly. However, little is known whether the DNA CG methylation profiles are increased with age in different population of leukocytes and involved in its gene expression and differentiation. This study addresses that knowledge gap.

We measured the DNA CG methylation profiles in a global genomic view of blood leukocytes from different age populations (0, 6, 20s, 40s and 60s) using a 1505 CG methylation array followed by identification of certain genes that hypermethylated more than 15% with aging. Moreover, we clarified the varied hypermethylation among different leukocyte populations, such as mononuclear cells (MNCs), polymorphonuclear leukocytes cells (PMNs) and T lymphocytes, and validated the leukocyte gene expression and differentiation related to manipulation of GSTM1 CG methylation.

## RESULTS

### Screening for DNA CG methylation genes in blood leukocytes of different age populations using a 1505 CG epigenetic array

**Figure 1:**
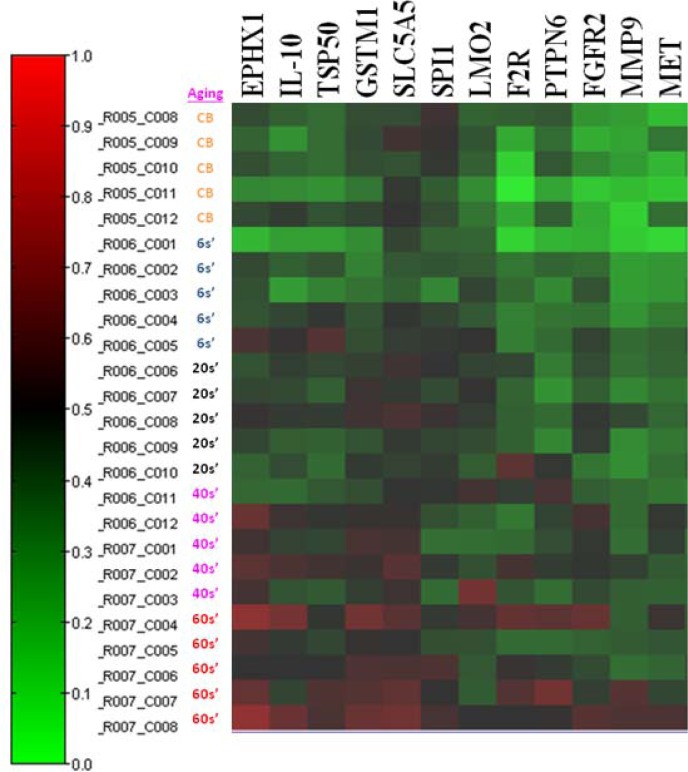
Screening for DNA CG methylation contents in blood leukocytes of different age populations using a 1505 CG methylation array A map of the methylation content change (≧15%) among blood cells of different ages (CB, neonatal cord blood; 6s', 5 to 6 years old; 20s', 20 to 30 years old; 40s', 40 to 50 years old; 60s', 60 to 70 years of age) derived from 5 replicate blood samples was presented, in which 12 CG sites in promotor or exon one regions had an increase in methylation content greater than 15% (EXHX1、IL-10、TSP50、GSTM1、SLC5A5、SPI1、F2R、LMO2、PTPN6、FGFR2、MMP9、MET). The red color represents higher methylation content.

To determine the DNA CG methylation genes in whole blood leukocytes of different age populations (0, 6, 20s, 40s, 60s) with five replicate samples, we used an epigenetic array to screen 1505 unique CpG sites located within the promoter and exon one regions of transcription coding sequence. As shown in Figure 1, we found that 12 genes (EXHX1、IL-10、TSP50、GSTM1、SLC5A5、SPI1、F2R、LMO2、PTPN6、FGFR2、MMP9、MET) revealed a hypermethylation greater than 15% CG methylation levels in promoter or exon sites as averaged from 5 replicate samples. Four genes (TRIP6、ABCB4、ABCC2、GML) had more than 15% decrease of the CG methylation. We then chose to validate the hypermethylation at promoter 266 CG site of the redox gene, GSTM1, and at exon one 128 site of the immune gene, LMO2, in different leukocyte populations of the different age groups.

### Validation of LMO2_E128 and GSTM1_P266 CG methylation in different population of leukocytes

**Figure 2:**
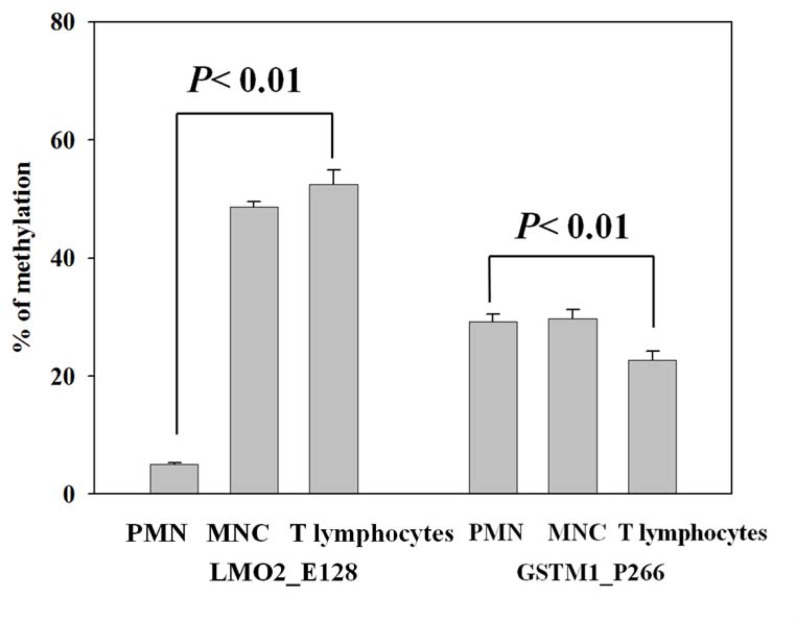
Validation of LMO2_E128 and GSTM1_P266 CG methylation in polymorphonuclear cells (PMNs), peripheral blood mononuclear cells (MNCs) and T lymphocytes Values are means± SE, n = 72. P values are determined as compared to PMN. Data were analyzed using one-way ANOVA followed by post hoc test.

We isolated peripheral whole blood, and separated different leukocyte populations for validation of the LMO2_E128 and GSTM1_P266 CG methylation in PMNs, MNCs and CD4 T lymphocytes. We found that the levels of LMO2_E128 CG methylation in T lymphocytes were the highest when compared with those of PMNs and MNCs (p< 0.01). In contrast, the levels of GSTM1_P266 CG methylation in CD4 T cells were lowest when compared with those of PMN and MNC cells (p< 0.01) (Figure 2 Left Panel, Right Panel). These results suggest that the levels of LMO2_E128 and GSTM1_P266 CG methylation in different populations of leukocytes were different.

### Validation of LMO2_E128 and GSTM1_P266 CG methylation of different population of leukocytes in different age populations

**Figure 3:**
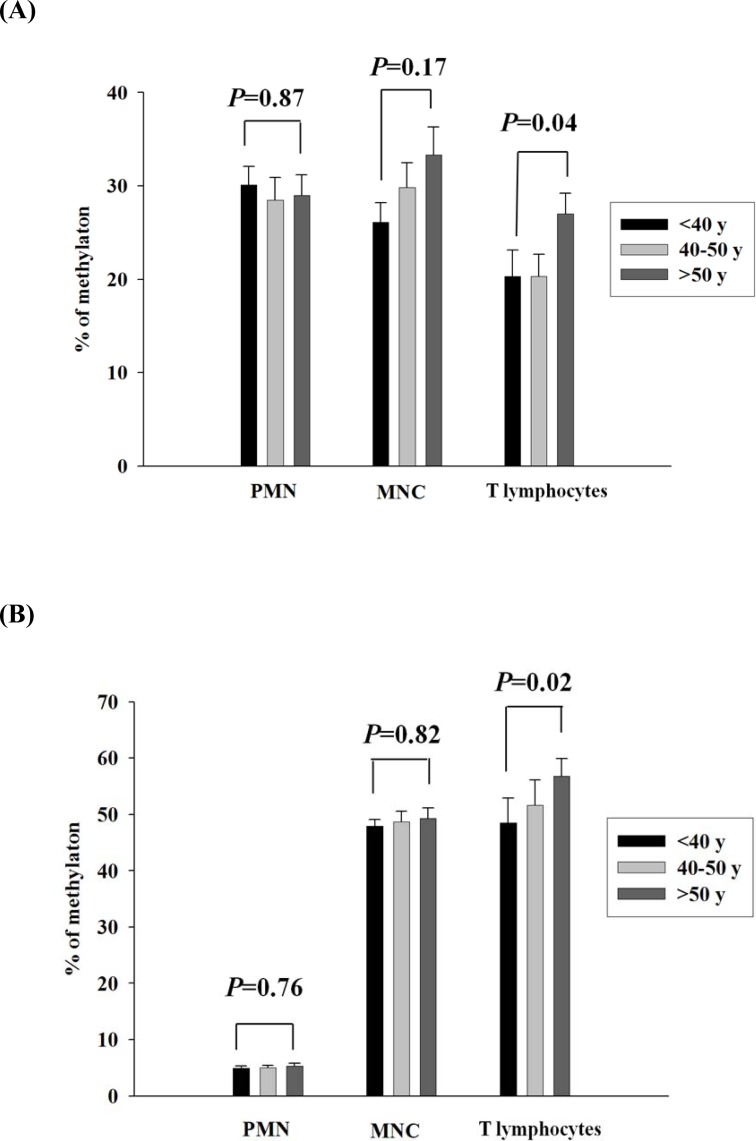
Validation of GSTM1_P266 and LMO2_E128 CG methylation of different population of leukocytes in different age populations A. The GSTM1_P266 CG methylation of polymorphonuclear cells (PMNs), peripheral blood mononuclear cells (MNCs) and T lymphocytes in different age populations. B. The LMO2_E128 CG methylation of PMNs, MNCs and T lymphocytes in different age populations. Values are means± SE, n = 72. P values are determined as compared to the under 40s group (< 40y) for different population of leukocytes. Data were analyzed using one-way ANOVA followed by post hoc test.

To further elucidate whether the CG methylation contents of LMO2_E128 and GSTM1_P266 in different populations of leukocytes increased with age, we next analyzed methylation levels of different populations of leukocytes in different age populations. We found that the levels of GSTM1_P266 and LMO2_E128 CG methylation in T lymphocytes but not those in PMNs and MNCs were significantly increased in the population older than 50 years of age when compared with T lymphocytes from those less than 40 years of age (p = 0.04 and p = 0.02, respectively) (Figure 3A, 3B). Results from this study indicate that the GSTM1_P266 and LMO2_E128 CG methylation contents of T lymphocytes increased with age over 50 years of age.

### Validation of LMO2 and GSTM1 mRNA expression of T cells in different age populations

**Figure 4:**
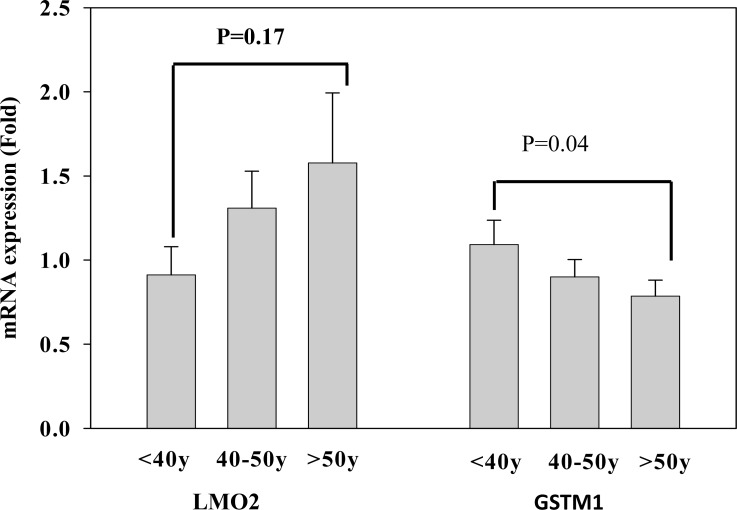
Validation of LMO2 and GSTM1 mRNA expression of T lymphocytes in different age populations (< 40, 40-50, and > 50 years of age), each group had 24 subjects Values are means± SE; P values are determined as compared to the under 40s group (< 40y). Data were analyzed using one-way ANOVA followed by post hoc test.

After identifying that GSTM1_P266 and LMO2_E128 CG methylation contents of T lymphocytes were increased with age, we further assessed whether the increase of CG methylation was correlated to decrease of LMO2 or GSTM1 mRNA expression. We used real time RT-PCR to validate LMO2 and GSTM1 mRNA expression of T lymphocytes in different age populations. Data revealed that the LMO2 mRNA expression of T lymphocytes increased with age, although the effect was not significant (p = 0.17). In contrast, GSTM1 mRNA expression of T lymphocytes in the population over 50 years of age was significantly decreased when compared to that in the population less than 40 years of age (p = 0.04) (Figure 4). Taken together, these results suggest that GSTM1, but not LMO2, mRNA expression in aged population decreased significantly related to higher GSTM1_P266 CG methylation.

### Validation of GSTM1 gene expression regulated by DNA methylation

**Figure 5:**
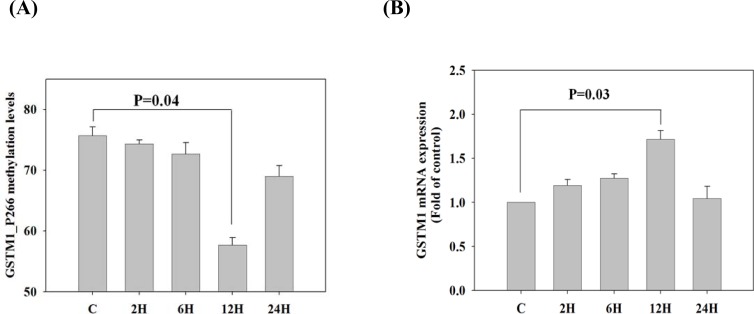
Validation of GSTM1 mRNA expression regulated by DNA methylation in Jurkat T cells A. The GSTM1_P266 CG methylation levels of Jurkat T cells stimulated by DNA methylation inhibitor (5-aza-2‘-deoxycytidine, 5 μM) for 2-24h. B. The GSTM1 mRNA expression of Jurkat T cells stimulated by DNA methylation inhibitor (5-aza-2‘-deoxycytidine, 5 μM) for 2-24h. Values are means± SE, n = 3. Data were analyzed using Kruskal-Wallis one-way ANOVA test.

To determine whether the GSTM1 mRNA expression was regulated by DNA methylation, we used DNA methylation inhibitor (5-aza-2‘-deoxycytidine, ADC) to validate the reciprocal changes between the promoter DNA methylation and mRNA expression of GSTM1 gene. We found that treatment of Jurkat T cells with ADC (5 μM) for 12 h significantly decreased GSTM1_P266 CG methylation levels (p= 0.004), when compared to the untreated group (Figure 5A). The decrease of GSTM1_P266 CG methylation by ADC was significantly associated with increase of GSTM1 mRNA expression (p = 0.03) (Figure 5B). The results suggest that the GSTM1 gene expression was regulated by GSTM1_P266 CG methylation in Jurkat T cells.

### Validation of GSTM1 mRNA expression involved in T helper cells differentiation

**Figure 6:**
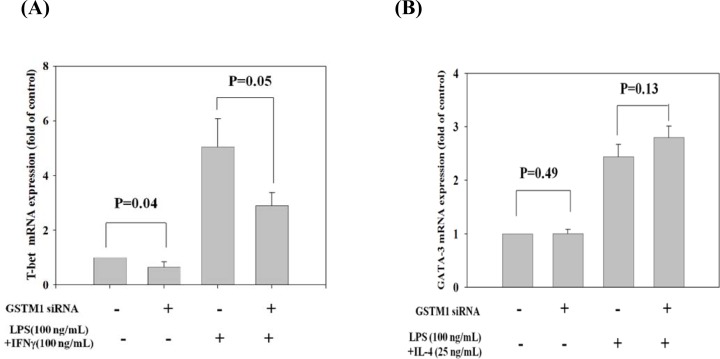
Effect of GSTM1 gene knockdown on Jurkat T cells differentiation as shown by T-bet and GATA-3 expression The Jurkat T cells were pre-treated with GSTM1 shRNA followed by stimulated with LPS (100 ng/mL) plus IFNγ (100 ng/mL) or IL-4 (25 ng/mL) for differentiation of T-bet A., and GATA-3 B. expression. GSTM1 hsRNA knockdown significantly decreased T-bet expression but not GATA-3 expression. Values are means± SE, n = 3. Data were analyzed by using Mann-Whitney U test.

**Figure 7:**
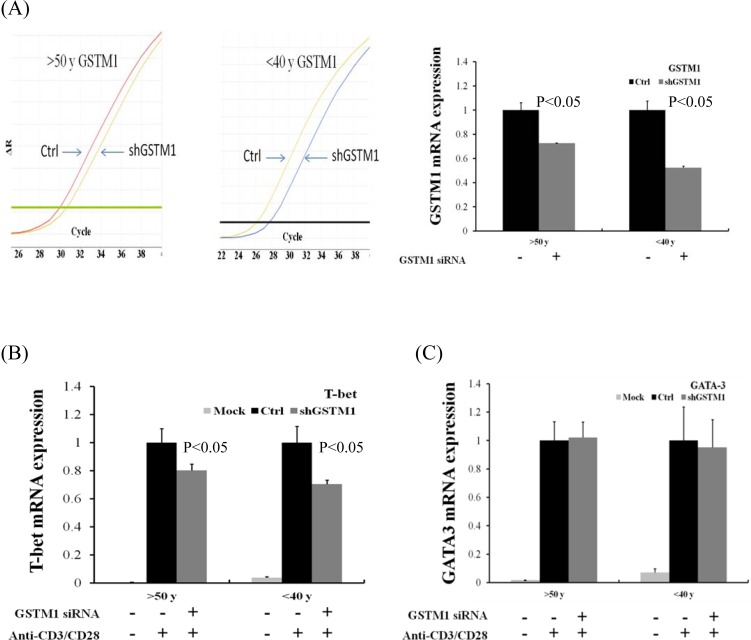
Effect of GSTM1 gene knockdown on CD4 T cells differentiation as shown by T-bet and GATA-3 expression The CD4 T cells purified by magnetic bead-based positive selection were pre-treated with GSTM1 shRNA for 24 hours, followed by anti-CD3 (10 ug/ml) and anti-CD28 (2 ug/ml) stimulation for 24 hours. A. shows a representative study of the hsGSTM1 gene knockdown both in older (>50 years of age) and younger (<40 years of age) adult CD4 T cells (left), and the data calculated from 3 experiments (right) showing significant downregulation of GSTM1 mRNA expression. The hsGSTM1 downregulation of GSTM1 mRNA was associated with a significant decrease in the Th1 but not Th2 polarization as shown by a significant decrease in T-bet expression B. but not a signigicant change of GATA-3 expression C. in older and younger adult CD4 T cells. Values are means± SE, n = 3. Data were analyzed by using Mann-Whitney U test.

We further demonstrated the effect of GSTM1 shRNA on the basal and LPS plus IFN-γ and IL-4 -induced type 1 T helper (Th1) and Th2 differentiation, as evidenced from the transcription factor T-bet and GATA-3 mRNA expression, respectively. We found that treatment of Jurkat T cells with GSTM1 shRNA for 24 h significantly decreased the basal T-bet mRNA expression (p = 0.04), as compared with that of untreated cells (Figure 6A), but the basal GATA-3 mRNA expression was not significantly changed (Figure 6B). We then pre-incubated Jurkat T cells with GSTM1 shRNA for 24 h followed by stimulation with LPS (100 ng/mL) plus IFN-γ (100 ng/mL) or IL-4(25 ng/mL) for 6h, respectively, and found that the T-bet mRNA expression of the GSTM1 shRNA treatment in Jurkat T cells was significantly decreased (p = 0.05), when compared with the untreated cell (Figure 6A). In contrast, the GSTM1 shRNA treatment did not affect the LPS plus IL-4 induced GATA-3 mRNA expression (Figure 6B). Additional studies with purified CD4 T cells from younger (<40 years of age) and older (>50 years of age) adults showed that GSTM1 gene knockdown by hsRNA could significantly (P<0.05) downregulate GSTM1 mRNA expression in 24 hours both in younger and older adult CD4 T cells (Figure 7A). The hsGSTM1 downregulation of GSTM1 mRNA caused a significant decrease in the Th1, but not Th2, polarization of CD4 T cells as shown by a significant decrease in T-bet expression but not a signigicant change of GATA-3 expression in younger and older adult CD4 T cells (Figure 7B and 7C). Examining cell proliferation, we found that the GSTM1 shRNA treatment for 6h or 24h did not affect Jurkat T cell or normal adult CD4 T cell proliferation (data not shown). Taken together, these results suggest that age-associated increase of GSTM1_P266 CG methylation is correlated to the decrease of GSTM1 mRNA expression and the decrease of Th1 differentiation as shown in decrease of the T-bet transcription factor expression.

## DISCUSSION

The main questions addressed by this study were: which gene′s CG methylation content increased or decreased accompanying with aging in a global genomic view of blood leukocytes from different age populations; whether the aging-associated increase of CG methylation levels were different among leukocyte populations and what function of the gene with aging-associated increase of CG methylation was changed. We did find that GSTM1_P266 CG methylation contents in T lymphocytes were increased in the older age population and that higher GSTM1 CG methylation was associated with the lesser GSTM1 mRNA expression and Th1 differentiation.

Several studies have previously determined age-associated epigenetic changes in primary tissues, such as saliva, dermis, epidermis [14], blood [15, 16] and cervical smear [17]. Aging-associated DNA-methylation changes are highly reproducible but most of them seem to have a general phenomenon. For instance, ten CpG sites were overlappingly identified with aging in skin, saliva and blood samples [2, 16, 18] Rakyan and colleagues [16] identified aging-associated differential methylation regions in whole blood, and then determined those in CD4+ T lymphocytes and CD14+ monocytes. They indicated that aging-associated differential methylation regions are consistent in whole blood leukocytes and could be used as a biomarker to predict aging and tissue degeneration. We, however, found that different leukocyte populations have different CG methylation contents in certain aging-associated CG methylation.

DNA hypermethylation was previously found in genes encoding for ribosomal DNA clusters as well as in those involved in DNA binding and regulation of transcription, leading to cardiovascular, respiratory and nervous system degeneration [19–21]. In this study, we found that the mRNA expression of GSTM1 in CD4 T lymphocytes decreased in the older age population, and was associated with increase of CG methylation content of GSTM1_P266. GSTM1 is mapped to the GST mu 1gene cluster on chromosome 1p13.3 genetic variant [22] that plays an important role in decreasing of oxidative stress and acts as a kind of antioxidant defense [23]. In addition, reduction of GSTM1 gene expression was associated with Parkinson's disease [24]. Moreover, GSTT1 and GSTM1 polymorphisms are associated with a risk to different cancers [25, 26] and other diseases related to oxidative stress [27]. We are the first in the literature to demonstrate that GSTM1 methylation levels increase with age, and are involved in Th1 cell differentiation. We also found that CG methylation levels of GSTM1_P266 were different in different populations of leukocytes. In particular, GSTM1_P266 CG methylation contents in T lymphocytes were increased with age and the GSTM1 mRNA expression reciprocally decreased with age. In addition, the GSTM1 gene expression was regulated by GSTM1_P266 methylation in Jurkat T cells. The dynamic control of the methylation contents were involved in the regulation of T helper cell differentiation as shown in Th1 transcription factor (T-bet) change.

An epigenetic basis for the helper T cell differentiation was based on the CpG methylation changes induced in the Ifng and Il4 loci as Th1 and Th2 cells, respectively, developed heritable patterns of cytokine gene expression [28, 29]. Previous study has indicated that the maintenance of methyltransferase gene had revealed derepression of cytokine expression during Th1 and Th2 maturation, which supported an important role for epigenetic effects on the organization of T helper cell differentiation [30]. Moreover, we found that expression of GSTM1 mRNA in Jurkat T cells was regulated by manipulation of DNA methylation and was implicated in T cells polarization to Th1 but not Th2 differentiation. Similarly, knockdown of GSTM1 mRNA expression in normal adult CD4 T cells also decreased the CD4 T cells polarization to Th1 but not Th2 differentiation. It is known that the dynamic control of T cell differentiation is regulated by the transcriptional machinery to gene regulatory regions, including promoters and enhancers [31], and that NF-E2-related factor 2 (Nrf2), another transcriptional factor, plays a significant immunomodulatory role in a number of models of inflammation [32]. In leukocytes, Nrf2 was reported to upregulate numerous genes, such as GSTM1, NAD(P)H:quinine oxidoreductase 1 (NQO1) [33], and heme oxygenase 1(HO-1) [34] that are useful markers of Nrf2 activation in leukocytes. Activation of Nrf2 may modulate cell-mediated immunity by repression of the Th1 cytokine IFNγ, while concurrently promoting the secretion of cytokines such as IL-4, IL-5, and IL-13 in wild-type CD4+ T cells. Besides, Nrf2 activation also suppresses T-bet DNA binding and promotes GATA-binding protein 3 DNA binding [35]. We demonstrated that GSTM1 promoter hypermethylation and decrease of its mRNA was involved in the reduction of T-bet expression.

The strength of this study is demonstrated by the screening of a large number (1505 CG sites) of aging-related hypermethylation sites in whole blood, followed by the validation with a different cohort of different leukocyte subpopulations among different age groups. There are a few limitations in the study. First, due to the high cost of genome-wide measurements of DNA methylation, we screened the methylation information from the 1505 CG sites representing genome-wide measurements of DNA methylation in five blood samples of 5 different age populations (0, 6, 20s, 40s and 60s). Second, we found 12 hypermethylated genes in the aged population but only validated two of the genes. Third, the sample size at 72 for the second cohort used to validate the CG methylation modulation of lymphocyte differentiation was relatively small. Fourth, we only validated the LMO2 and GSTM1 expression on the Th1 and Th2 polarization of Jurkat T cells and purified adult CD4 T cells but not other T cell populations. In the future experiments, we may need to study the epigenetic modulation of LMO2 and GSTM1 functions on CD8 T cells since Tserel, et al. [36] have recently reported a strong inverse relationship between CG methylation contents and gene expression levels of T cells, showing 10 genes in CD4 T cells and 272 genes in CD8 T cells revealed reciprocal changes between CG methylation and gene expression. Fifth, we have validated the methylation contents of GSTM1_P266 in T lymphocytes were significantly higher in aged population associated with lower mRNA expression. This, however, did not exclude any additional DNA methylation sites in GSTM1 that may be even more relevant to regulate GSTM1 expression and immune functions. Therefore, our results require another larger population of validation before interpreted as the whole view of epigenetic profiles of aging immunity.

In summary, the present study demonstrates that 12 genes' promoter or exon 1 CG methylation contents were increased by more than 15%, but 4 genes' promoter or exon 1 CG methylation decreased by more than 15% in the blood leukocyte of older aged population. We are the first to validate that GSTM1_P266 and LMO2_E128 CG methylation contents in T lymphocytes, but not PMNs or MNCs, were significantly increased in the aged population. Lower GSTM1 CG methylation content leads to the higher GSTM1 mRNA expression in T cells and knockdown of GSTM1 mRNA expression decreases T cells polarization to Th1 differentiation. GSTM1 genetic polymorphisms have been implicated in many diseases including atherosclerosis [37], and bladder cancer [38]. We are the first to show epigenetic control of the aging-dependent GSTM1 promoter methylation on suppression of the redox GSTM1 gene expression and lymphocyte T cell differentiation. This warrants the search for small-molecule inhibitors targeting key epigenetic changes as immunomodulatory drugs for the cell immunity of older people.

## MATERIALS AND METHODS

### Chemicals and reagents

RPMI-1640 medium, fetal bovine serum (FBS), penicillin, L-glutamine, and nonessential amino acids (NEAA) were purchased from Gibco/BRL (MD). 5-aza-2‘-deoxycytidine as an inhibitor of DNA methylation was purchased from Sigma-Aldrich (St. Louis, Missouri, USA) and dissolved in acetic acid: water (1:1) at appropriate concentrations. Lipopolysaccharide, and human recombinant IFN-γ and IL-4 expressed in E. coli were obtained from Sigma-Aldrich (St. Louis, Missouri, USA) and dissolved in phosphate buffered saline (PBS) at appropriate concentrations.

### Profiling of genome-wide CpG methylation of leukocytes from different age populations

The GoldenGate Methylation BeadChip (Illumina, San Diego, CA, USA) was used to perform genome-wide screening of DNA methylation patterns among leukocyte DNA age group samples from neonatal cord blood (0 year of age), 6s (5 to 6 years of age), 20s (20 to 30 years of age), 40s (40 to 50 years of age) and 60s (60 to 70 years of age). The DNA samples of neonatal cord blood and children 6 years of age were obtained from the decoded DNA samples of a birth cohort study [39]; the DNA samples of healthy adults in 20s (20 to 30 years of age), 40s (40 to 50 years of age) and 60s (60 to 70 years of age) were collected in the Center for Physical Evaluation of the study hospital after informed consent was obtained. To minimize a diverse environmental exposure of the participants studied, we collected the DNA samples in one month and subjected to initial screening of DNA methylation profiles. The GoldenGate Methylation panel targets 1505 unique CpG sites located within the proximal promoter regions of transcription start sites and exons sites of coding sequencing (CDS) in the NCBI Database [40]. DNA samples (500 ng) isolated from identification-decoded blood sample were subjected to bisulfite conversion of unmethylated CG sites to UG sites using the EZ DNA Methylation Kit (Zymo Research Corporation, Irvine, CA, USA) according to the manufacturer's instructions. The bisulphite-treated DNA was PCR amplified to form biotin-labeled, single-stranded PCR products by PyroMark PCR kit (Qiagen, Hilden, Germany). Assays for the selected candidate CpG sites GSTM1_P266 and LMO2_E128 were designed using the pyrosequencing Assay Design 2.0 software (Qiagen, Valencia, CA). The PCR products were sequenced using PyroMark Q24 Pyrosequencer (Qiagen, Valencia, CA) after verifying the positive PCR products by visualizing the appropriately sized band on an 1% agarose gel. The relative methylation levels were quantitated by PyroMark Q24 software (Qiagen, Valencia, CA). In the genome-wide CG site methylation array analysis, the genes showing more than 15% increase or decrease of CG site methylation levels between different age populations were chosen for further analysis based on previous reports in which a 5-17% difference in CG methylation level was chosen for validation [41–46].

### Validation of age-associated hypermethylation in different leukocyte populations

Whole blood samples were collected from 72 adult individuals, including 24 participants less than 40 years old (group 1), 24 participants between 40 and 50 years old (group 2) and 24 participants more than 50 years old (group 3). There were 21 males and 51 females in the total sample. All the clinical specimens were untagged and re-labeled by numbers as sample identifiers. The polymorphonuclear cells (PMNs) and peripheral blood mono-nuclear cells (MNCs) were separated from whole blood by density gradient centrifugation. The whole bloods were mixed with 4.5% dextran solution in PBS, and then the mixture set for 30 minutes at room temperature. The upper layer containing the leukocyte-rich fraction was recovered and then layered onto 2.5 mL of Ficoll-Paque Plus (GE Healthcare, Sweden) in a 15 mL tube. The tube was centrifuged at 400 × g for 30 minutes. The leukocyte populations could be separated into 4 parts: PMNs and the red blood cell pellet (called the lower layer), the Percoll phase, the MNCs in middle layer, and the plasma in upper layer. After hypotonic lysis of remaining erythrocytes, PMNs were isolated by centrifugation (100 × g for 10 min) in the lower layer pellet, and the middle MNCs layer was washed twice with PBS and resuspended with PBS [47]. In order to study modulation of lymphocyte gene expression and differentiation related to DNA methylation, we isolated the CD4+ T lymphocytes from MNCs by using the human CD4+ selection kit for cell separation with AutoMACS cell separator (Miltenyi) [48]. The purity and viability of the PMNs, MNCs and CD4 lymphocytes were greater than 95% and 98%, respectively.

### Real-time polymerase chain reaction for analysis of transcription factor mRNA expression

The total RNA samples of PMNs, MNCs and T lymphocytes were extracted by Trizol reagent (Life Technologies, Grand Island, NY, USA), followed by isopropanol precipitation. Total RNA concentration was determined using infinite M200 Pro Spectrophotometer (TECAN, Grödig, Austria). The cDNA was generated from 500 ng of total RNA using Superscript III First-stand Synthesis Super Mix for real-time PCR kit with 25 ng Random Hexamers (Invitrogen, California, USA) according to the manufacturer's instructions. Real-time quantitative polymerase chain reaction (Q-PCR) analysis was performed using the ABI 7500 fast real-time system (Applied Biosystems, Marsiling, Singapore), and the Power SYBR Green PCR Master Mix (ABI, Warrington, UK). For each sample, GAPDH expression levels as determined by threshold cycles (CT) were used for normalization purposes. Each gene expression was computed as the difference (ΔCT) between the target gene and house-keeping gene, GAPDH, CT values, as our previously described [49]. The ΔCT method was then applied to calculate the mRNA quantification determined by the equation at 2-ΔCT. The primer sequences for the mRNA expression of LMO2 and GSTM1 genes are set as follows:

LMO2 forward primer: 5′-GGACCCTTCAGAGGAACCAGT-3′;

LMO2 reverse primer: 5′-GGCCCAGTTTGTAGTAGAGGC-3′;

GSTM1 forward primer: 5′- GGGACGCTCCTGATTATGACA-3′;

GSTM1 reverse primer: 5′- AAGTAGGGCAGATTGGGAAAGTC-3′;

GAPDH forward primer: 5′-CATGAGAAGTATGACAACAGCCT-3′;

GAPDH reverse primer: 5′-AGTCCTTCCACGATACCAAAGT-3′.

### Modulation of T cell differentiation toward type 1 and type 2 T helper reactions by GSTM1 expression

We used human Jurkat T cells line as a model to determine T cell differentiation [50]. The Jurkat T cells were obtained from the Food Industry Research and Development Institute (FIRDI, Taiwan) and maintained in RPMI-1640 medium supplemented with 10% fetal bovine serum, 100 IU/ml penicillin/streptomycin, and 2 mm L-glutamine under 5% CO2 at 370C. The shRNA carrying puromycin selection marker was purchased from the National RNAi Core Facility of Academic Sinica (Taiwan). The sequence used for targeting GSTM1 was as follow: GSTM1: 5′- CGTTCCTTTCTCCTGTTTATT -3′. Cells were transfected with the shRNA plasmid and the stable clone with the highest knockdown efficiency were used for further studies. Jurkat T cells were pretreated with GSTM1 shRNA for 24 h and then incubated with LPS (100 ng/mL) plus IFNγ (100 ng/mL)/ IL-4(25 ng/mL) for 6 h. Similarly, purified CD4 T cells from younger and older adults were subjected to GSTM1 shRNA knockdown for 24 h before stimulation for the assessment of T cell Th1 and Th2 polarization. The total RNA of the cells was extracted by Trizol reagent (Life Technologies, Grand Island, NY, USA), followed by isopropanol precipitation. The transcription factor expression of T helper (Th) cell polarization toward Th1 or Th2 was quantitated by Q-PCR analysis of T-bet or GATA-3 expression, respectively, as our previously described [49]. The primer sequences for the mRNA expression of T-bet and GATA-3 genes are set as follows:

T-bet forward primer: 5′-GATGTTTGTGGACGTGGTCTTG-3′;

T-bet reverse primer: 5′-CTTTCCACACTGCACCCACTT-3′;

GATA-3 forward primer: 5′-GCG GGC TCT ATC ACA AAA TGA-3′;

GATA-3 reverse primer: 5′-GCT CTC CTG GCT GCA GAC AGC-3′;

GAPDH forward primer: 5′-CATGAGAAGTATGACAACAGCCT-3′;

GAPDH reverse primer: 5′-AGTCCTTCCACGATACCAAAGT-3′.

### Data management and statistical analysis

In the initial screening of leukocyte DNA methylation profiles, we measured and used the same amount of DNA samples (500 ng) obtained from whole blood leukocytes for comparison of DNA CG-methylation profiles. The methylation contents of the CG sites with 15% incease or decrease among different age groups were subjected to validation of the differences in different purified leukocytes. In the validation of the target gene CG methylation including LMO2 and GSTM1, DNA samples of purified PMNs, MNCs and CD4 T cells (purity > 95%) from different age groups were normalized and subjected to measurement of CG methylation contents. Similarly, RNA samples from purified PMNs, MNCs and CD4 T cells (purity > 95%) from different age groups were subjected to RT-PCR detection of mRNA expression in normalization with internal control GAPDH expression. Data from this study were expressed as means ± SE and analyzed using one-way ANOVA followed by post hoc test using the Statistical Package for Social Sciences (SPSS Inc., Chicago) version 17.0 for Windows. Statistical significance was set at a p value of £ 0.05.

## Abbreviations

MNCs: mononuclear cells
PMNs: polymorphonuclear leukocytes cells
Th1: type 1 T helper cells
ADC: 5-aza-2‘-deoxycytidine
FBS: fetal bovine serum
NEAA: non-essential amino acid
PBS: phosphate-buffered saline
IRB: institutional review board
